# An AI-ready remote sensing dataset for high-resolution forest disturbance mapping

**DOI:** 10.1038/s41597-026-07084-8

**Published:** 2026-03-26

**Authors:** Enmanuel Rodríguez-Paulino, Johannes Stoffels, Martin Schlerf, Achim Röder, Alexander Wagner, Thomas Udelhoven

**Affiliations:** 1 Remote Sensing and Natural Resources Modelling Group, Luxembourg Institute of Science and Technology (LIST), Belvaux, 41, rue du Brill, Luxembourg, L-4422 Germany; 2 Earth Observation and Climate Processes, Trier University, Behringstr. 21, Trier, 54286 Germany; 3 Forschungsanstalt für Waldökologie und Forstwirtschaft, Landesforsten Rheinland-Pfalz, Hauptstraße 16, Trippstadt, 67705 Germany

## Abstract

Europe’s forests face increasing threats from natural disturbances such as insect outbreaks, pathogens, and windthrow, often aggravated by extreme weather events and followed by subsequent salvage logging. Monitoring these events at high spatial detail is essential for forest management and climate adaptation, yet many remain undetected when using medium-resolution satellite imagery, and manual reporting by authorities is time-consuming and inconsistent. Here we present a high-resolution, deep learning-ready dataset designed for the classification of forest disturbance types. It consists of ~17,500 image patches (500 × 500 pixels at 0.2 m resolution) derived from digital orthophotos of Rhineland-Palatinate, Germany. Each patch includes five channels (red, green, blue, near-infrared, and object height) and a segmentation mask with labeled disturbance classes such as bark beetle damage, clear-cuts, and windthrow. To demonstrate its utility, we apply a deep learning model and assess the contribution of individual channels through ablation analysis. The model achieved an overall accuracy of 88.2%, with near-infrared and object height identified as the most informative channels. The dataset offers a high-resolution resource for advancing deep learning-based forest disturbance monitoring.

## Background & Summary

Forests in Germany, like many in Central Europe, have experienced increasing tree mortality over the past decade^1^. This trend is driven by a combination of droughts, storms, insect and disease outbreaks, wildfires, along with human interventions such as salvage logging, and infrastructure development^2–6^. Forest disturbance mapping has traditionally been carried out using medium-resolution (10–30 m) satellite imagery^7^. While medium-resolution wall-to-wall remote sensing maps provide valuable insights into disturbance regimes, they systematically underestimate the extent of small-scale or fragmented disturbances^8,9^, and often fail to disentangle spatially overlapping events^10^. These shortcomings are particularly limiting in the heterogeneous and fragmented landscapes of European forests, where disturbances frequently occur in small forest stands (~0.05 ha) that are ecologically important but often missed by medium-resolution sensors^11–14^. Addressing these gaps requires data sources capable of capturing small and irregularly shaped patches with higher spatial fidelity.

In many regions of Germany, forest health monitoring relies primarily on *in-situ* data, which serves as the source for large-scale forest disturbance assessments. This information is often complemented by digital orthophotos (DOP), high-resolution aerial imagery acquired from aircraft, and additional forest information layers provided by forest administrations. DOP enable local foresters to visually inspect disturbed areas and integrate image interpretation with their knowledge of local forest conditions. However, this process is time-consuming, susceptible to human bias, and dependent on consistent data recording and annotation criteria across individuals.

High-resolution imagery can address the spatial limitations of medium-resolution data and provides an important basis for automating the manual recording of disturbances. Although acquisition is costly, high-resolution aerial data are now frequently available in many countries (e.g., Belgium^15^, Czechia^16^, Finland^17^, France^18^, Germany^19^, Latvia^20^, Luxembourg^21^, Poland^22^, Spain^23^, and Switzerland^24^) at resolutions of 0.1–1 m, often free of charge. This open-access initiative, following the European legislation on open data^25^, has spurred research that leverages multispectral and multi-temporal DOP data to map forest disturbances with unprecedented spatial detail. For instance, canopy-level forest mortality has been successfully mapped using multispectral (RGB-NIR) DOP from Germany, Luxembourg, and Finland^26–28^. Another recent initiative, deadtrees.earth^29^, provides access to very high-resolution aerial imagery for mapping dead standing wood. However, available annotations are typically community-contributed, optional, primarily focused on individual dead-standing trees, and they are not optimized for deep learning workflows. Despite these advances, the number of open-source, multispectral DOP-based datasets that are well-suited for training deep learning models to detect diverse forest disturbance types remains limited.

Deep learning methods have recently emerged as powerful tools for analyzing high-resolution remote sensing imagery, enabling the detection of diverse forest disturbance events as evidenced by the increasing number of research articles on this topic. Architectures such as U-Net, a convolutional neural network (CNN) variant, have improved the accuracy of classification and segmentation tasks by capturing multiscale contextual information related to forest canopies and disturbances^30^. U-Net has proven highly effective in mapping bark beetle infestations^31^, windthrow events^32^, tree mortality^27,33^, and fire damage^34^ across various data types and spatial scales. However, despite outperforming traditional machine learning methods in complex tasks^35^, the lack of reliable ready-to-use training data often limits their widespread adoption^36^. To address this gap, we introduce the Deep Learning for Forest Disturbance mapping (Deep4Dist) dataset, a novel semantic segmentation resource developed in Rhineland-Palatinate (RLP), Germany, and designed to support disturbance mapping across similar forest ecosystems in Central Europe. This dataset comprises over 20,000 disturbance records distributed across approximately 17,500 georeferenced image patches, each represented as a 500 × 500-pixel image at a spatial resolution of 0.20 m (quadratic patches of 100 m side length). Each image contains four spectral and one structural channel: red, green, blue, near-infrared (NIR), and object height, offering a rich multidimensional representation of forest conditions. The dataset’s potential applications are demonstrated using a U-Net-based deep learning framework, offering groundwork for further research into automated forest disturbance monitoring.

## Methods

### Study area

The Deep4Dist dataset was collected over the forested area of RLP, a federal state in western Germany. RLP has the highest forest coverage in the country, with forests occupying approximately 43% of its total area^37^. Dominant tree species include European beech (*Fagus sylvatica*), sessile oak (*Quercus petraea*), pedunculate oak (*Quercus robur*), Scots pine (*Pinus sylvestris*), and Norway spruce (*Picea abies*).

Ecologically, RLP forms part of the Continental biogeographical region^38^, which spans much of central and eastern Europe and accounts for nearly one-third of the European Union’s territory.

**Fig. 1:**
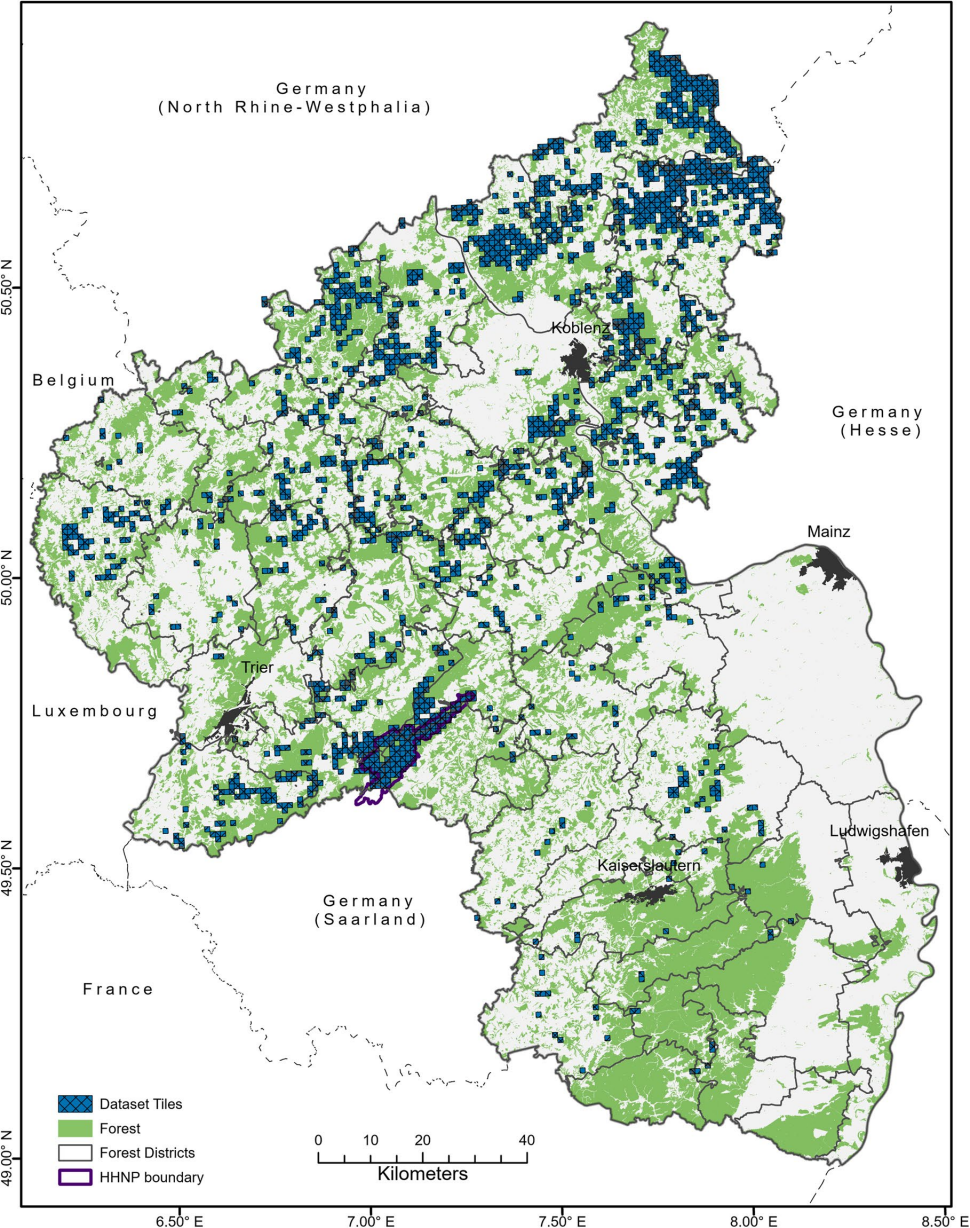
Spatial distribution of all image tiles in the Deep4Dist dataset containing annotated forest disturbances across Rhineland-Palatinate, Germany. Each square represents a 500 × 500-pixel image patch.

Forest health in RLP has declined over the past four decades, with the 2024 regional crown condition survey reporting 87.5% of assessed trees classified as damaged^39^. Norway spruce was the most affected species, with an area loss of more than 20% between 2012 and 2022^40^. The geographic location of the study area and the spatial extent of the dataset are shown in Fig. 1.

### Data sources

#### Aerial imagery

The aerial images are high-resolution DOP acquired annually across RLP at a spatial resolution of 0.20 m^41^. These orthophotos, comprising four spectral bands (blue, green, red, and NIR), are publicly available under open license (https://geoshop.rlp.de/dl-de_by-2-0.html) and can be accessed through the RLP GeoShop website (https://www.geoportal.rlp.de). Due to the alternating flight campaign strategy, 50% of the state is surveyed each year, resulting in temporal coverage of individual locations every two years.

#### Object height (OBH)

This layer was generated by subtracting a digital elevation model (DEM) from a digital surface model (DSM). The DSM was derived from photogrammetric point clouds generated during the same aerial survey campaigns as the digital orthophotos, at a spatial resolution of 0.20 m^42,43^, ensuring temporal consistency between spectral and surface data. The DEM represents the bare-earth surface and was derived from aerial laser scanning campaigns with varying acquisition dates across the RLP region and is delivered at a spatial resolution of 1 m^44^. The DEM was assumed to be temporally invariant for the purpose of object height estimation. Prior to subtraction, the DEM was resampled to 0.20 m using bilinear interpolation and co-registered to the DSM grid. Both datasets, the DEM and DSM, are distributed under the same open license and website as the DOP. The object height layer was then computed as DSM minus DEM. This structural layer complements the spectral information and enhances the discrimination of forest disturbance types by incorporating vertical canopy structure.

#### Ground truth labels

The labels used to annotate the dataset were obtained from two primary sources: the Hunsrück-Hochwald National Park (HHNP) disturbance database and the Digital Forest Protection Reporting System (DFPR). The HHNP database, maintained since 2016, is a point-based disturbance database collected on the ground by rangers in the areas where human-intervention has been temporarily permitted during a transitional phase within the national park. The records include the geographic coordinates, disturbance information and forest stand attributes. In contrast, the DFPR database records forest disturbances across RLP with both point and polygon geometries since 2019 and is administered by the State Forest Administration in RLP (Landesforsten Rheinland-Pfalz). However, DFPR polygons, manually annotated by local foresters, often represent the boundaries of forest stands rather than the actual disturbance extents. This discrepancy necessitated the use of a model-assisted approach to refine the polygon geometries, ensuring alignment with the physical extents of disturbances. Both databases contain observations regarding bark beetle infestations, windthrow, snow damage, tree removals, and the disturbance onset.

### Dataset creation

#### Image preparation

To prepare the input data, the RGB-NIR orthophotos were stacked with the corresponding OBH layer to form a 5-channel image. Each DOP scene, originally sized at 10,000 × 10,000 pixels, was divided into non-overlapping patches of 500 × 500 pixels (equivalent to 100 m side length). This tiling approach standardizes the patch size while avoiding the need for additional padding or mosaicking. The resulting dataset comprises image tiles with dimensions of 500 × 500 × 5 (height × width × channels), with channels ordered as R, G, B, NIR, and OBH. All image tiles intersecting at least one annotated forest disturbance polygon were retained in the dataset.

#### Manual and semi-automatic annotation

**Fig. 2:**
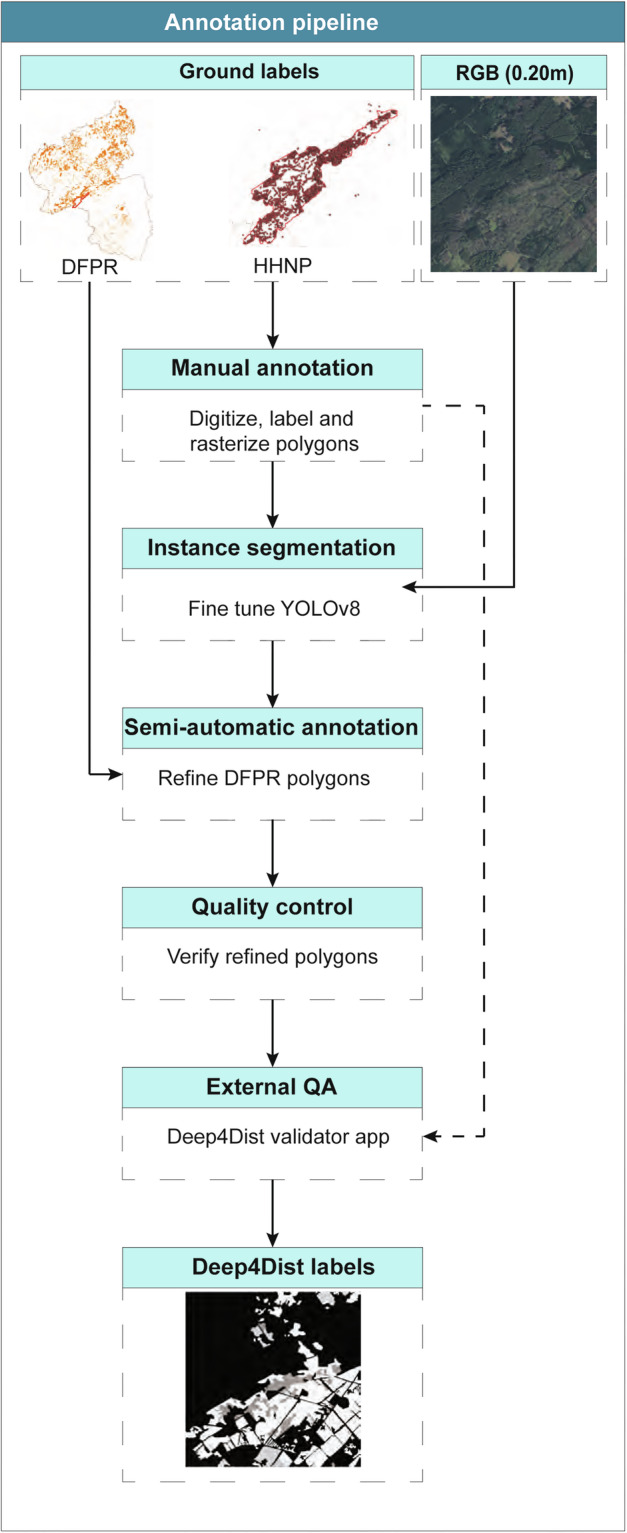
Pipeline used to annotate the Deep4Dist dataset. The workflow combines manual annotation with labels from the HHNP database, refines DFPR polygons using YOLOv8, and includes quality control and external quality assessment with the Deep4Dist Validator app.

The annotation process followed a two-stage pipeline combining manual delineation and deep learning-based refinement. In the first stage, disturbances within HHNP were manually digitized as polygons through photointerpretation of a bi-annual time series of very high-resolution aerial imagery (0.20 m spatial resolution, 2015–2023). Each polygon was assigned a disturbance type using metadata from the HHNP disturbance database, ensuring that labels corresponded to verified field observations. These manually curated annotations served as the ground truth training data to fine-tune a YOLOv8 instance segmentation model^45^. In the second stage, the fine-tuned YOLOv8 model was then applied to RGB DOPs to predict disturbance extents within DFPR polygons. Model outputs were used to adjust polygon boundaries by aligning them with the visible spatial extent of disturbed areas, as identified from aerial imagery. Boundary adjustments were guided by visual agreement between model predictions, and DFPR metadata to ensure consistency and accuracy. Finally, both sets of annotations, the manually delineated disturbances within the HHNP and the DFPR-refined disturbance polygons, were merged to produce the final annotated Deep4Dist dataset. This hybrid approach streamlined the annotation process while maintaining high-quality standards. Figure 2 depicts the pipeline used to annotate the segmentation masks in the dataset.

#### Disturbance classes definition

The Deep4Dist dataset includes three forest disturbance classes (bark beetle damage, clear-cuts, and windthrow) and one background class derived from the combination of the ground truth labels and the semi-automatic annotation process. It is worth noting that all class definitions are derived specifically from the top-view perspective provided by aerial imagery.

- Bark beetle: areas with standing trees exhibiting progressive discoloration, visible in RGB-NIR channels. This definition was applied only in locations where bark beetle infestations were reported in the reference databases and, where available, supported by multi-temporal image interpretation.
- Clear-cuts: areas of complete or selective logging where no standing trees or with exposed root plates remain.
- Windthrow: includes uprooted or fallen trees, often with attached root plates. This class captures both recently fallen green trees and those previously killed by bark beetle infestation.
- Background: all pixels not belonging to the defined disturbance classes, including disturbances smaller than the minimum mapping unit of 100 m^2^.

**Fig. 3:**
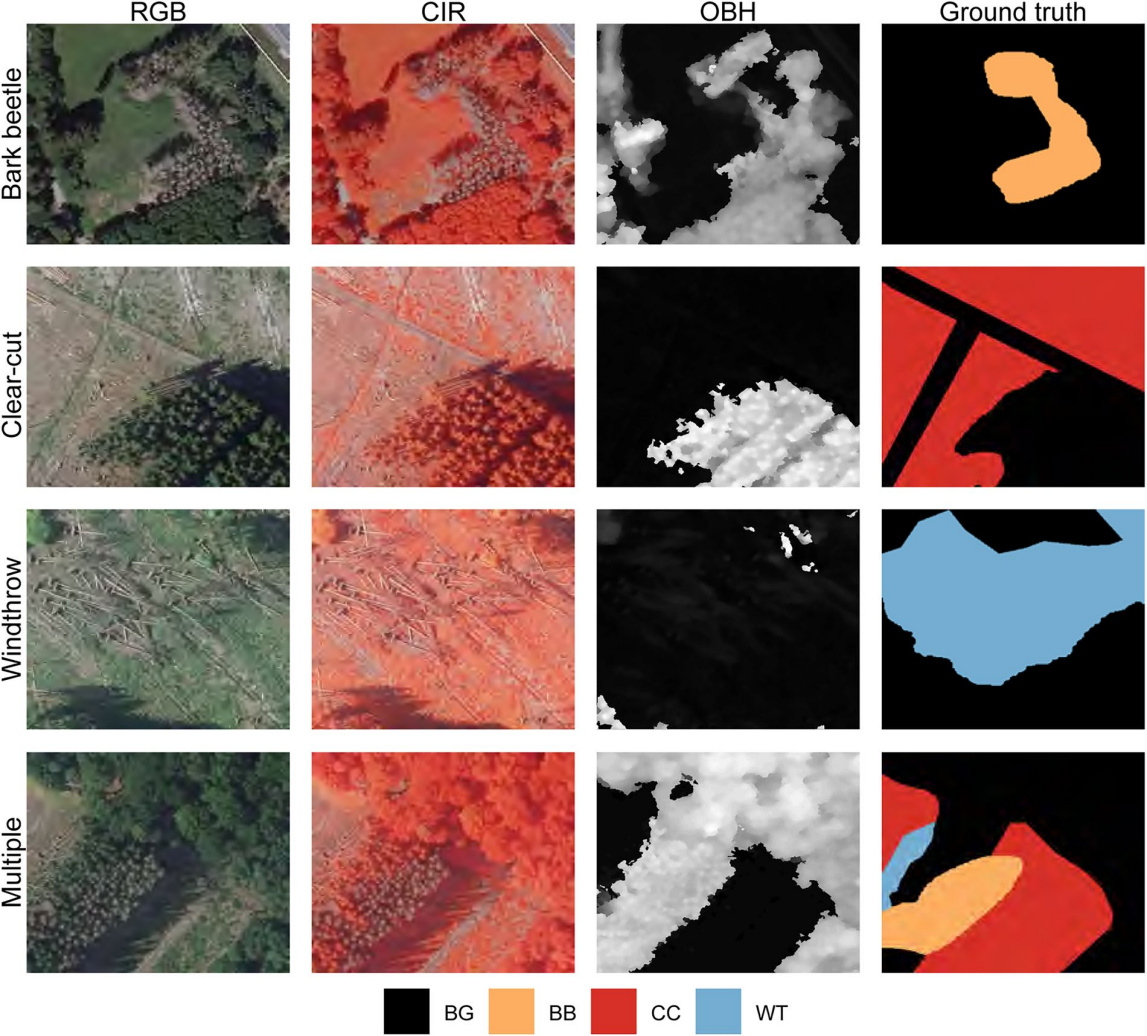
Examples of forest disturbance classes and input data layers in the Deep4Dist dataset. Rows show example patches for the disturbance categories: bark beetle damage, clear-cut, windthrow, and areas affected by multiple temporally co-occurring disturbances. Columns display, from left to right, the natural-color (RGB) composite, color–near-infrared (CIR) composite, object height (OBH) layer, and the corresponding ground truth masks. In the ground truth column, bark beetle damage is shown in orange, clear-cut in red, windthrow in blue, and background in black.

Disturbance class distributions in the dataset are imbalanced, with bark beetle damage (9408 records) and clear-cuts (9851 records) well represented, while windthrow events (1269 records) are relatively underrepresented. Figure 3 illustrates example patches of each disturbance class.

#### Dataset partitioning

For the demonstration exercise, the dataset was split into training, validation, and testing subsets based on the administrative boundaries of forest districts within RLP. Each image patch was assigned to a partition according to the location of its center point, ensuring that the sets are spatially disjoint. This strategy minimizes the risk of overestimation due to spatial autocorrelation.

Detailed statistics regarding the number of disturbance records and image patches per subset are presented in Table 1. The dataset was split into 70% for training, 25% for validation, and 5% for testing.

**Table 1:** Data partition sizes and forest disturbance event statistics.

Partition	Disturbance	No. Records	No. Patches	Total Patches
Train	Bark beetle	6397	5579	12147
	Clear-cut	6746	5883	
	Windthrow	786	685	
Validation	Bark beetle	2511	2051	4470
	Clear-cut	2546	2080	
	Windthrow	415	339	
Test	Bark beetle	500	401	905
	Clear-cut	559	449	
	Windthrow	68	55	

A record represents the individual disturbance observations while patches refer to 100 m by 100 m tiles in the dataset. Note that patch counts are not mutually exclusive across disturbance types, as individual image tiles may contain multiple disturbance classes.

## Data Records

**Fig. 4:**
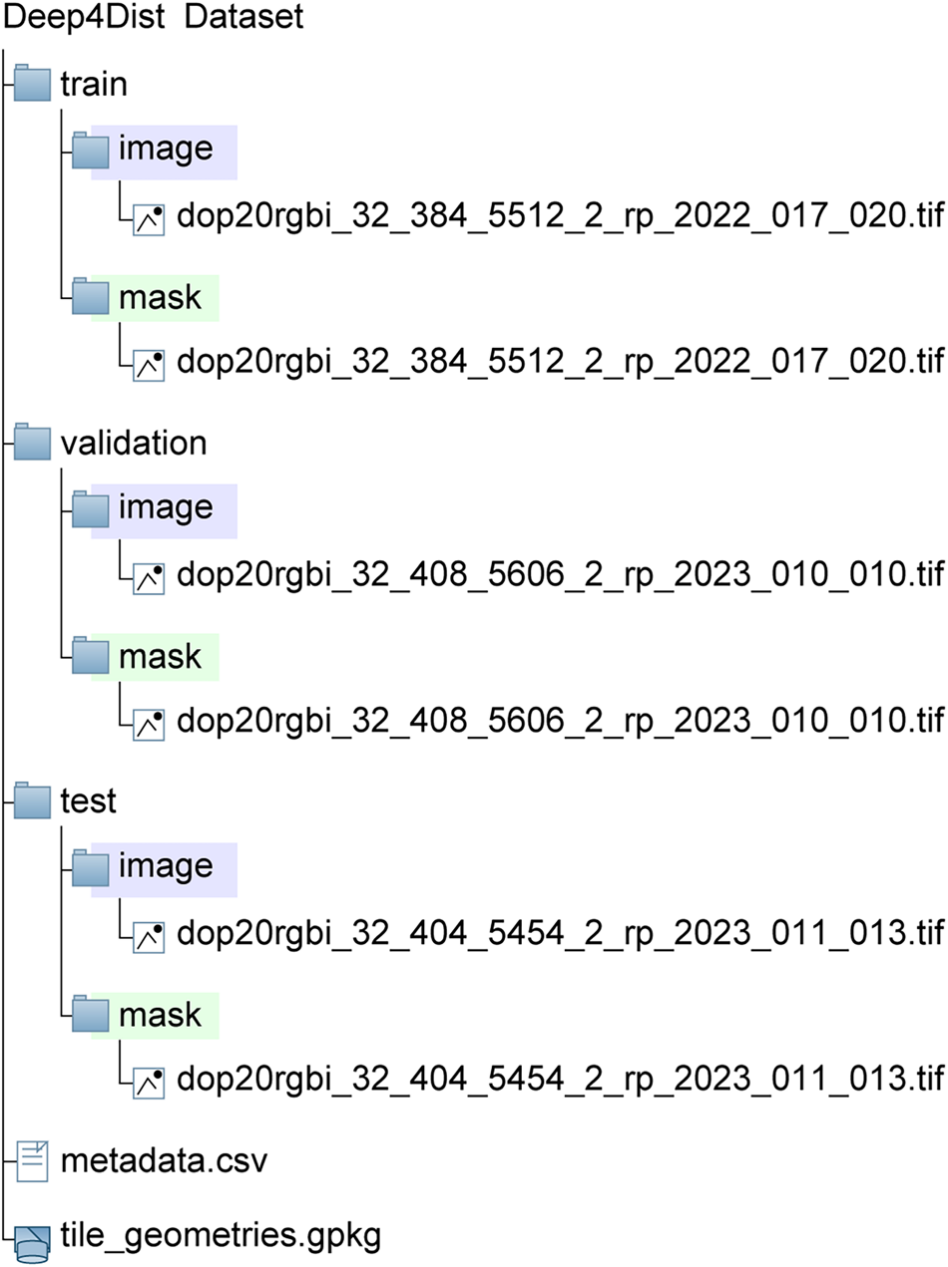
Schematic directory tree of the Deep4Dist dataset, showing the train, validation, and test set folders with their image and mask subfolders, plus the metadata files.

The Deep4Dist dataset is publicly available on Zenodo at 10.5281/zenodo.14884818 and is distributed under the CC BY 4.0 license^46^. The repository includes separate folders for the training, validation, and test sets. Within each set, data are organized into two directories: image and mask. The image directories contain multilayer imagery (RGB, NIR, and OBH), while the mask directories contain the corresponding semantic segmentation masks. To facilitate reproducibility, the dataset is distributed with the same splits used in our experiments. Nevertheless, users may define alternative partitions to better suit their specific applications. Figure 4 illustrates the structure of the repository.

All image files are delivered in GeoTIFF format and are georeferenced using the European Terrestrial Reference System 1989 (ETRS89) and the Universal Transverse Mercator (UTM) projection Zone 32 North (EPSG code 25832), ensuring full compatibility with the original DOP coordinate system. Additionally, the dataset includes a metadata file that lists, for each patch, a unique identifier, the coordinates of its center, the acquisition date, and the disturbance agent. A supplementary GeoPackage file is also provided, which delineates the geographic extent of each image tile. This allows users to rapidly visualize the dataset in GIS software and perform spatial queries.

Each image patch is named following a structured convention derived from the original DOP product. A filename is structured as follows: “dop20rgbi_32_404_5454_2_rp_2023_011_013.tif”; this filename includes several components: (i) dop20rgbi, referring to the original DOP product spatial and spectral resolution from which the patch was derived; (ii) 32, 404 and 5454, indicating the UTM zone (32) and the leading three- and four-digit coordinates of the scene’s northern and eastern bounds, respectively; and (iii) rp_2023, which encodes metadata related to the acquisition campaign, including region code, and year; 011_013 corresponds to the internal Deep4Dist patch identifier. Although the prefix “dop20rgbi” does not explicitly mention the OBH band, every image patch in Deep4Dist includes the OBH layer as a fifth band. We have retained the original naming convention for consistency with the source data.

## Technical Validation

### Dataset characteristics

**Fig. 5:**
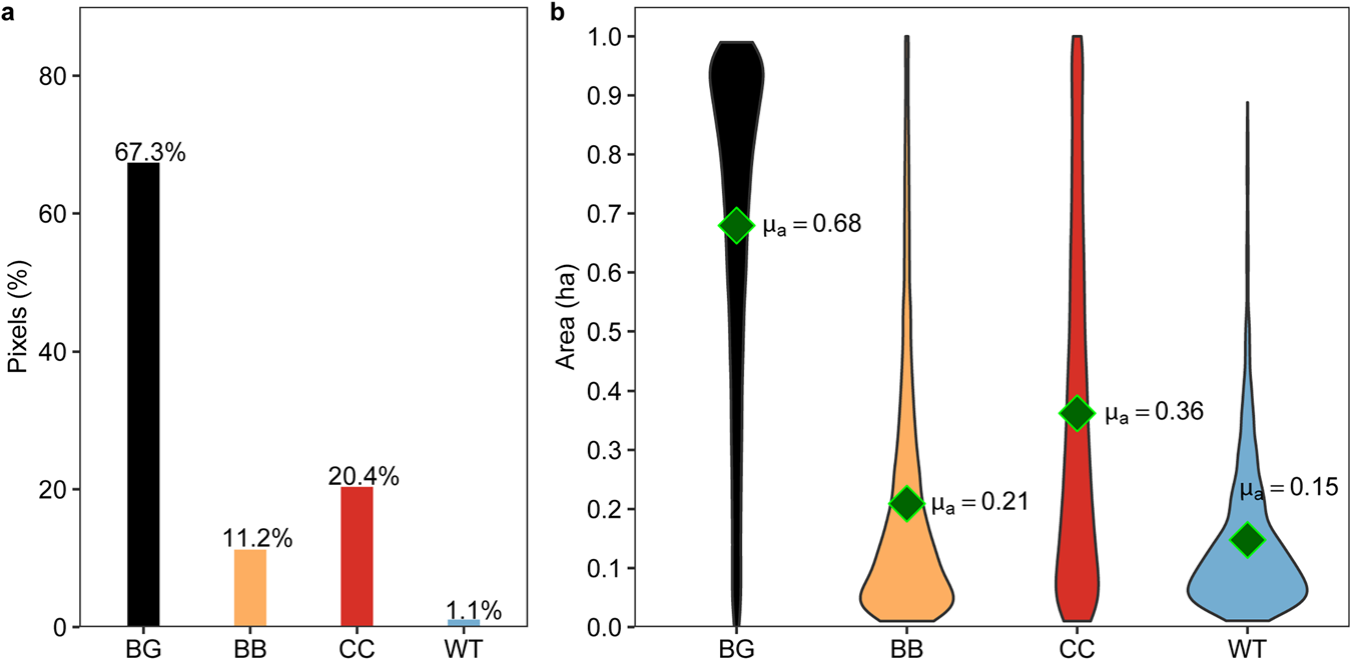
Distribution of pixels (percentage) per class (**a**), and total area (hectares) occupied by a given class within individual image tiles (**b**). Green dots indicate mean area values.

In the Deep4Dist dataset, background pixels account for 67.3% of all labeled pixels, while disturbed pixels represent 32.7%, as depicted in Fig. 5. Among disturbance classes, clear-cuts comprise the largest share of disturbed pixels (20.4%), followed by bark beetle damage (11.2%) and windthrow (1.1%). Mean class-wise area per tile varies across classes, with background areas showing the largest average extent (µ_a_ = 0.68 ha), followed by clear-cuts (µ_a_ = 0.36 ha), bark beetle damage (µ_a_ = 0.21 ha), and windthrow (µ = 0.15 ha). At the image-tile level, disturbed areas cover on average 27.9% of a tile (SD = 26.0%), reflecting substantial spatial heterogeneity across the dataset.

### Quality assessment

To assess the quality of the annotations described, a team of remote sensing specialists with expertise in vegetation monitoring and machine learning conducted a visual inspection of approximately 5,500 image patches and their corresponding segmentation masks. The inspected samples were randomly selected from the training, validation and test sets. Evaluators compared each segmentation mask to its associated aerial image and approved it only if the delineated disturbance accurately reflected the type and extent of the visible feature. Masks failing to meet this standard were initially rejected during the quality assessment. However, rejected tiles were not excluded from the dataset. Instead, each rejected patch was systematically re-examined and corrected where necessary, including adjustments to instance boundaries, class labels, or the addition of missing disturbance objects. After correction and internal verification, these tiles were reintegrated into the final Deep4Dist dataset. Each image patch was independently reviewed once.

The assessment was performed remotely using the Deep4Dist Dataset Validator App (https://enmrodpau.shinyapps.io/Deep4Dist_Validator), a web-based platform developed specifically for this task. The external quality assessment yielded a 92% acceptance rate, indicating high annotation accuracy and supporting the dataset’s suitability for scientific and operational use. Evaluators could include comments justifying their decisions, which were subsequently categorized and are summarized in Table 2. Some comment categories reflect minor or negligible issues that allowed for acceptance, while others point to errors in boundary delineation, class labeling, or overall data quality, warranting rejection.

**Table 2:** Definition of comment categories used in the quality assessment of the dataset.

Comment category	Definition	Decision
Perfect Match	Instances accurately represent both the boundaries and class of the phenomenon.	Accepted
Minor Acceptable Inaccuracy	Instances generally represent the phenomenon, with only slight inaccuracies in boundaries.	Accepted
Rejected Without Comment	The tile was excluded from analysis without further explanation.	Rejected
Boundary Inaccuracy	Instance boundaries do not align accurately with the real-world disturbed area.	Rejected
Incorrect Class Label	The instance is labeled with an incorrect class, not corresponding to the actual phenomenon.	Rejected
Missing Object	Disturbed areas present in the image are not annotated.	Rejected
False Positive	An instance was labeled as disturbed, but the area is actually undisturbed.	Rejected
Annotation Policy Disagreement	The annotation does not follow the defined class decision policy.	Rejected
Noisy Input	The input image is optically unclear or degraded, affecting annotation accuracy.	Rejected

Each category describes a type of observation made during quality control and is classified as either leading to acceptance or rejection of the patch based on annotation accuracy and adherence to labeling guidelines.

Table 3 shows the distribution of expert comments across dataset splits. Most annotations were classified as perfect matches or accepted with minor inaccuracies. A smaller fraction was rejected due to issues such as incorrect class labeling or boundary inaccuracies.

**Table 3:** Expert comment distribution across dataset splits.

Comment category	Dataset split	Total tiles
Perfect match	Train	3151
Minor acceptable inaccuracy		410
Rejected without comment		177
Boundary inaccuracy		38
Incorrect class label		32
Missing object		29
False positive		8
Annotation policy disagreement		7
Perfect match	Validation	401
Minor acceptable inaccuracy		57
Rejected without comment		29
Missing object		3
Boundary inaccuracy		2
Noisy input		1
Perfect match	Test	61
Rejected without comment		13
Minor acceptable inaccuracy		5

#### Benchmarking

To demonstrate the dataset’s technical quality and ecological relevance, an initial benchmarking was conducted using a ResU-Net34 model. Rather than developing a new model, the goal was to show that the high-resolution, multispectral, and structural information in Deep4Dist enables robust classification of forest disturbances. We report both overall and class-specific performance metrics.

### Model details

The forest disturbance classification problem was formulated as a semantic segmentation task addressed by a CNN. The U-Net architecture^47^ with a ResNet34 encoder^48^ was selected for its proven efficacy in remote sensing applications, particularly for forest-related applications^27,49,50^. Model weights were initialized using transfer learning from a ResU-Net34 trained on the French Land cover from Aerospace Imagery (FLAIR) dataset^51^. FLAIR shares spatial, spectral, and geographic characteristics with Deep4Dist (RGB, NIR, and OBH bands at 20 cm resolution), making it a strong candidate for domain-adapted transfer learning.

### Training details

The training pipeline was implemented in Python using PyTorch. We normalized the input images using the 10% and 90% quantile values for each channel. We applied standard data augmentation methods, including random 90° rotations, vertical and horizontal flips. Additionally, we applied simple random channel dropout (zeroing out 2 input bands at a time). This augmentation mitigates sensitivity to distribution shifts that arise when input bands are removed during post-training ablation analyses. The full training parameters are summarized in the supplementary Table 1. The training set comprised 12,147 images processed in mini-batches of 32, while 4,470 images were reserved for validation and processed in mini-batches of 16. Each image patch consisted of five input bands (RGB, near-infrared, and object height).

### Evaluation metrics

Model performance was evaluated on 905 test images using overall accuracy (OA), macro F1 (F1), and macro intersection over union (IoU), providing complementary measures of classification accuracy, class balance, and spatial overlap.

### Input channel ablation

We performed an input channel ablation analysis by systematically zeroing out individual and paired input bands at inference time. By observing the degradation in metrics, such as F1, when certain bands were omitted, we inferred the relative importance of each band in the segmentation process. Importantly, feature ablation also addresses real-world scenarios where input datasets may lack certain bands, such as NIR or OBH channels.

### Experimental results

The model achieved an overall accuracy of 88.2%, a macro F1 score of 81.9%, and a macro IoU of 70.3% on the test set, closely aligning with the external expert quality assessment’s acceptance rate of over 92%. Class-wise performance metrics are reported in Table 4. The background, bark beetle, and clear-cut classes showed strong performance, while windthrow exhibited lower scores, likely reflecting its lower representation in the dataset and higher structural variability.

**Table 4:** Class-wise metrics using the best-performing model.

F1	IoU	Disturbance
91.0	83.5	BG
86.5	76.3	BB
83.2	71.3	CC
66.8	50.2	WT

**Fig. 6:**
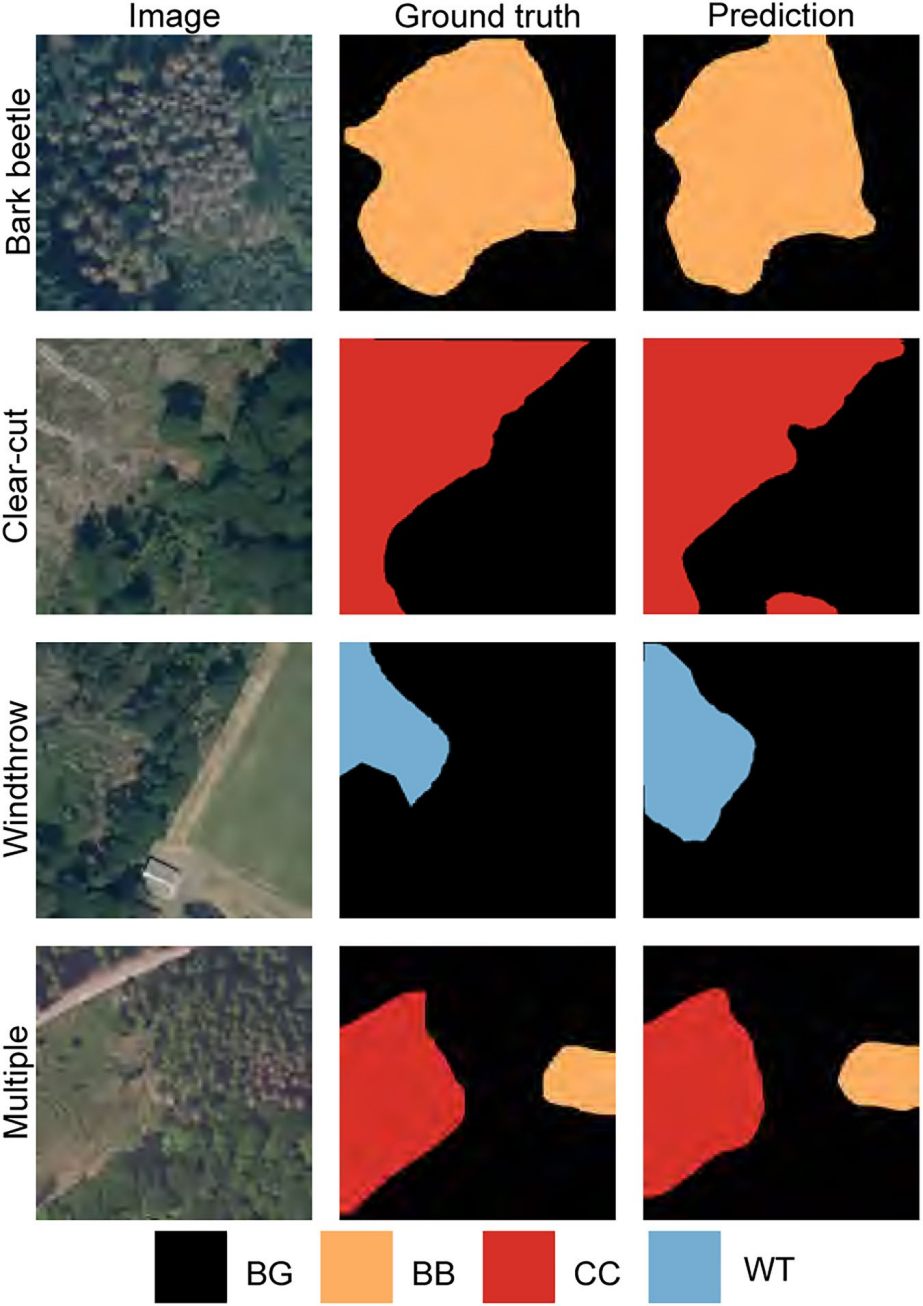
Example model predictions compared with ground truth masks. Each row corresponds to a disturbance category: bark beetle damage, clear-cut, windthrow, and multiple temporally co-occurring disturbances. Columns show, from left to right, the RGB image, the ground truth mask, and the corresponding model prediction. In the mask panels, bark beetle damage is shown in orange, clear-cut in red, windthrow in blue, and background in black.

Figure 6 illustrates qualitative examples of the model’s performance on test patches. Overall, predictions closely match the spatial extent and shape of disturbed areas, with minor discrepancies primarily occurring along object boundaries. The model trained on the Deep4Dist dataset achieves performance levels consistent with previously published deep learning–based studies focusing on individual disturbance types, such as bark beetle and windthrow^31,32,52,53^.

**Fig. 7:**
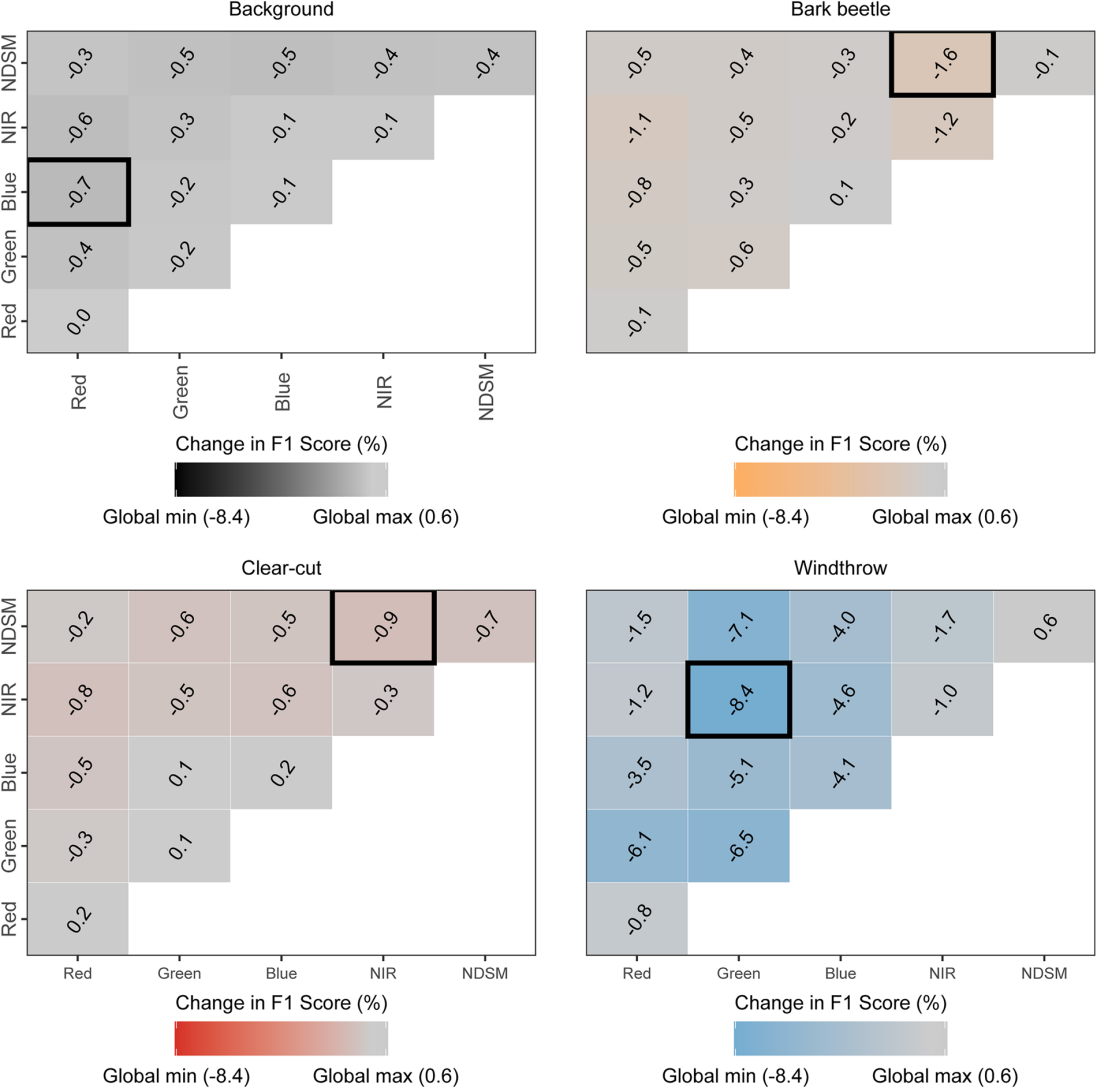
Impact of zeroing out bands on F1. Heatmaps show the percentage change in F1 when specific bands are removed compared to the best performing model. The black box indicates the largest drop in F1 in each panel.

Figure 7 shows the percentage change in F1 for each disturbance class when bands are zeroed out. Despite the use of random channel-drop augmentation during training, some bands still have a large impact on predictive performance, illustrating their ecological importance in monitoring specific forest disturbance types. In particular, bark beetle and clear-cut detection were most sensitive to the removal of NIR and object height information, while windthrow showed the strongest performance decrease when green and near-infrared bands were jointly removed. These results highlight the complementary role of spectral and structural information in supporting robust forest disturbance mapping.

## Usage Notes

### Dataset usage

The Deep4Dist dataset provides a valuable resource to remote sensing researchers, forest ecologists, and forest authorities by offering expert-annotated, high-resolution, deep learning–ready data that enables detailed mapping of multiple forest disturbances, and supports the development and validation of robust deep learning semantic segmentation models.

### Limitations

Although the dataset represents a substantial part of the European continental ecoregion, differences in forest dynamics, ecological conditions, and sensor characteristics may affect downstream analyses. Users applying it beyond its original spatial, temporal, or sensor context should consider including region-specific reference data, and validation against local observations. Finally, undisturbed samples were not included in the Deep4Dist dataset, as the reference databases only document disturbance events and do not provide validated labels for undisturbed forest conditions.

## Supplementary information

Supplementary Information

## Data Availability

All files in the Deep4Dist dataset are available at 10.5281/zenodo.14884818 and is distributed under the CC BY 4.0 license.

## Code availability

The source code is openly available on GitHub (https://github.com/enmanuelrodpau/deep4dist) and includes scripts for creating, preprocessing, and analyzing the dataset, as well as for benchmarking and input channel ablation experiments.
