# Supplementary material for: An AI-ready remote sensing dataset for high-resolution forest disturbance mapping
Source: Sci Data. 2026 Mar 26;13:490. doi: 10.1038/s41597-026-07084-8 (PMC13031536; doi:10.1038/s41597-026-07084-8)
Supplement: Supplementary file 1 — Supplementary Information [file 41597_2026_7084_MOESM1_ESM.pdf]

Supplementary Table 1: Model Hyperparameters and library setups used during the dataset benchmarking.

**Section**	**Parameter**	**Value**	**Library**
Model	Architecture	U-net	Segmentation-models-pytorch
	Encoder	ResNet34	
Augmentation	RandomChannelDrop	p=0.5	Kornia
	HorizontalFlip	p=0.5	
	VerticalFlip	p=0.5	
	RandomRotation	p=0.5, degree=90	
Data Normalization	Quantile 10% (RGBIE)	15, 26, 24, 75, 0	N/A
	Quantile 90% (RGBIE)	162, 158, 148, 239, 186	
Class weights	Background	0.1	N/A
	Bark beetle	0.25	
	Clear-cut	0.25	
	Windthrow	0.4	
Training	Epochs	100	Pytorch,pytorch-lightning
	Early stopping patience	50	
	Batch size	32	
	Deterministic mode	warn	
Optimizer config	Learning rate	0.0001	Pytorch,pytorch-lightning
LR scheduler	Factor	0.1	Pytorch,pytorch-lightning
	Mode	minimum	
	Value to monitor	validation loss	
	Patience	3	
Computation	Seed	2024	Pytorch,pytorch-lightning
	No. GPUs	8	

Version
0.3.3
0.7.3
N/A
N/A
2.4.0,2.1.1
2.4.0,2.1.1
2.4.0,2.1.1
2.4.0,2.1.1
